# Faricimab in the Treatment of Retinal Disease: A Narrative Review

**DOI:** 10.7759/cureus.114098

**Published:** 2026-08-06

**Authors:** Mahmoud Zeaiter, Hadil Jaber, Koussay Gharbi, Shiona Maria Benedict Fernandes, Jawad Mchaik, Mohamed Ahmed, Amir Hassan, Ahmad A Hammoud

**Affiliations:** 1 Ophthalmology, New Vision University, Tbilisi, GEO; 2 Medicine, Ilia State University, Tbilisi, GEO; 3 Surgery, Ivane Javakhishvili Tbilisi State University, Tbilisi, GEO; 4 Medicine, Caucasus International University, Tbilisi, GEO; 5 Medicine, David Tvildiani Medical University, Tbilisi, GEO; 6 Medicine, European University, Tbilisi, GEO

**Keywords:** diabetic macular edema, faricimab, neovascular age related macular degeneration, polypoidal choroidal vasculopathy, retinal disease, retinal vein occlusion

## Abstract

Retinal and choroidal diseases, including diabetic macular edema, neovascular age-related macular degeneration, retinal vein occlusion, and polypoidal choroidal vasculopathy, remain a significant cause of visual impairment around the world, especially among elderly . Although anti-vascular endothelial growth factor (anti-VEGF) therapy remains the standard of care, it is limited by its side effect profile, resistance, and high injection burden. This underscores the need for a more optimal and possible replacement. Faricimab is an emerging bispecific antibody targeting VEGF-A and angiopoietin-2, addressing both angiogenesis and vascular permeability by modulating the tyrosine kinase with immunoglobulin-like domains-2 pathway. This literature review synthesizes information from preclinical studies and randomized controlled trials. It focuses on the molecular reasoning behind the use of faricimab and the safety profile compared with anti-VEGF monotherapy.

## Introduction and background

Chorioretinal eye diseases mostly affect the elderly, with increased incidence in patients older than 60 years of age, and patients with prior cataract surgery, diabetes mellitus, and systemic hypertension being at increased risk [1,2]. Different treatment modalities have been developed to manage retinal eye diseases, targeting their diverse underlying causes and disease mechanisms [3]. For instance, the first-line treatment for diabetic macular edema (DME) is anti-vascular endothelial growth factor (anti-VEGF) therapy, which targets vascular leakage but is limited by a high recurrence rate and a significant percentage of patients who experience persistent edema, demanding frequent injections. Alternative treatments, such as corticosteroids, offer anti-inflammatory benefits but carry other adverse effects such as cataracts and glaucoma; if retinal disease is not controlled early, irreversible losses in visual acuity and vision loss often ensue [4]. Other chorioretinal disorders such as polypoidal choroidal vasculopathy (PCV) show incomplete or low rates of regression with anti-VEGF agents and frequently persist or recur despite repeated treatment [5].

Faricimab, a bispecific antibody targeting VEGF-A and angiopoietin-2 (Ang-2) used to treat retinal diseases, when compared to anti-VEGF therapy, allows for extended dosing intervals in a significant proportion of patients, which reduces treatment burden without compromising efficacy [3,6]. These results are supported by studies showing improved outcomes in patients treated with retinal eye diseases (DME, neovascular age-related macular degeneration (nAMD), retinal vein occlusion (RVO), and PCV) than standard anti-VEGF therapy [7-9].

This review will explain the molecular mechanisms behind faricimab and its effectiveness compared with other treatment modalities for retinal eye diseases, including DME, nAMD, RVO, and PCV.

## Review

Molecular reasoning behind faricimab

Anti-VEGF monotherapy has been the gold standard treatment for many retinal diseases, including nAMD [10-12]. Evidence indicates that anti-VEGF monotherapy is often insufficient to achieve optimal therapeutic outcomes [7]. This could be partially explained by the fact that the pathophysiology of these diseases is multifaceted, including a range of biological processes [13,14]. This complexity highlights the need for a multitargeted therapeutic course to achieve better outcomes [7].

VEGF is a proangiogenic factor expressed in various pathological conditions in response to tissue hypoxia and hypoperfusion, which can be caused by retinal vascular damage or obstruction [15]. Its expression is upregulated by the hypoxia-inducible factor 1, eventually leading to abnormal angiogenesis and alterations in the vasculature such as increased vascular permeability [15,16].

Angiopoietin-1 (Ang-1) and the transmembrane receptor tyrosine kinase with immunoglobulin-like domains-2 (Tie2), expressed by the retinal endothelial cells, are key regulators in retinal homeostasis [17,18]. Ang-1 binds to Tie2, activating a signaling pathway that promotes vascular stability and controls vascular permeability [17,19]. Ang-2, an Ang-1 antagonist, is upregulated in various retinal vascular diseases [17-19]. By displacing Ang-1 from its receptor, Ang-2 mitigates the vasoprotective effects of the Ang-1/Tie2 signaling pathway [17,18]. Ang-2 further amplifies the actions of VEGF, leading to excessive fibrosis, increased vascular permeability, abnormal angiogenesis, and inflammation [18,19]. Furthermore, Ang-2 exerts direct proangiogenic effects independent of its Tie2 signaling pathway, destabilizing the vasculature and inducing apoptosis of the pericytes through an integrin-mediated pathway [20].

Faricimab is a 150 kDa bispecific monoclonal antibody that binds to both VEGF and Ang-2 [21,22]. Its molecular structure is comprised of three distinct domains: an antigen-binding fragment directed against Ang-2, another against VEGF, and a modified fragment crystallizable (Fc) region [21]. The Fc region was specifically engineered to prevent its binding to both the neonatal Fc receptor and Fc-gamma receptor, thereby minimizing systemic distribution and limiting its proinflammatory signaling [15]. By concurrently targeting both Ang-2 and VEGF, faricimab demonstrates prolonged therapeutic durability by promoting vascular stabilization [23]. Dual inhibition of VEGF and Ang-2 suppresses choroidal neovascularization more efficiently than inhibition of either pathway alone in preclinical studies [15].

Research comparing the anatomic outcomes of using faricimab vs. aflibercept (a VEGF inhibitor) concluded that faricimab was superior to aflibercept in achieving faster resolution of retinal fluid and greater reduction in central subfield thickness (CST), a key biomarker in retinal disease activity in patients with nAMD [20]. Administration of faricimab every 16 weeks provided treatment durability equivalent to aflibercept administered every eight weeks at week 48 in these patients [24]. Moreover, the intravitreal injection of faricimab in patients with DME with a treat-and-extend personalized interval regimen improved best corrected visual acuity (BCVA) equally to aflibercept after two years of follow-up [25].

Phase 2 clinical trials show that faricimab is more effective than ranibizumab (VEGF-A inhibitor) in improving CST and BCVA in DME [26]. A study by Khanani et al. investigated the use of 6 mg of intravitreal faricimab at 12- or 16-week intervals, or 0.5 mg of intravitreal ranibizumab every four weeks, in patients with nAMD at 40 weeks, achieving both visual and anatomical outcomes comparable with ranibizumab while requiring fewer injections [27]. Furthermore, those results were sustained through 53 weeks with faricimab administered at extended intervals matching the durability of monthly ranibizumab [27].

In addition to its equivalent and more durable treatment efficacy, treatment with faricimab may reduce the treatment burden associated with anti-VEGF therapy by enabling extended dosing intervals in appropriate patients, thereby reducing the frequency of intravitreal injections and the associated psychological and financial burden [15]. Intravitreal administration of anti-VEGF agents, including faricimab, carries procedure-related risks such as endophthalmitis, retinal detachment, ocular hypertension, and other injection-related complications. Faricimab's extended dosing intervals may reduce cumulative exposure to these risks by lowering the number of injections required [28].

Faricimab in the management of retinal diseases

Faricimab has shown significant results in the treatment of retinal diseases by targeting both VEGF-A and Ang-2 together. This section will review the clinical evidence supporting its efficacy across major exudative retinal disorders.

Diabetic Macular Degeneration

DME develops through VEGF-A-driven vascular leakage combined with Ang-2-mediated inflammation and vessel instability. The efficacy of Faricimab’s dual pathway inhibition is supported by results from the BOULEVARD phase 2 trial, where faricimab demonstrated greater visual acuity gains than ranibizumab [26].

The pivotal YOSEMITE and RHINE phase 3 trials supported the mechanistic hypothesis. Over a two-year period, a personalized treat-and-extend protocol with faricimab demonstrated visual acuity (BCVA) gains that were noninferior to those achieved with fixed-interval aflibercept dosed every eight weeks. In the YOSEMITE and RHINE trials, a treat-and-extend approach was used, meaning the time between injections was adjusted depending on how the patient's eye responded. The idea is to initiate treatment with loading doses and then gradually extend the interval between injections according to disease activity. If the retina looks stable, the interval is extended, and if fluid or activity comes back, the interval is shortened. This way, patients get the right amount of treatment without too many injections, which can save visits and reduce risk, while keeping vision under control. Using this method, many patients were able to reach 12- or even 16-week intervals, showing that Faricimab can be both effective and durable [7]. An important finding regarding durability was observed over two years: 61%- 64% of pooled faricimab-treated patients were able to extend treatment intervals to at least 12 weeks, and 42%-46% achieved the maximum 16-week interval [7]. Faricimab produced greater reductions in CST than aflibercept [29]. Faricimab also resulted in a higher proportion of patients achieving complete resolution of macular leakage on fluorescein angiography [8]. The results of these trials are in line with previous studies and serve to validate the effectiveness of faricimab in treatment-naïve patients and those resistant to other anti-VEGF agents [30,31]. Hence, the evidence shows that faricimab is more beneficial than visual results while also decreasing the number of intravitreal injections required.

**Figure 1:**
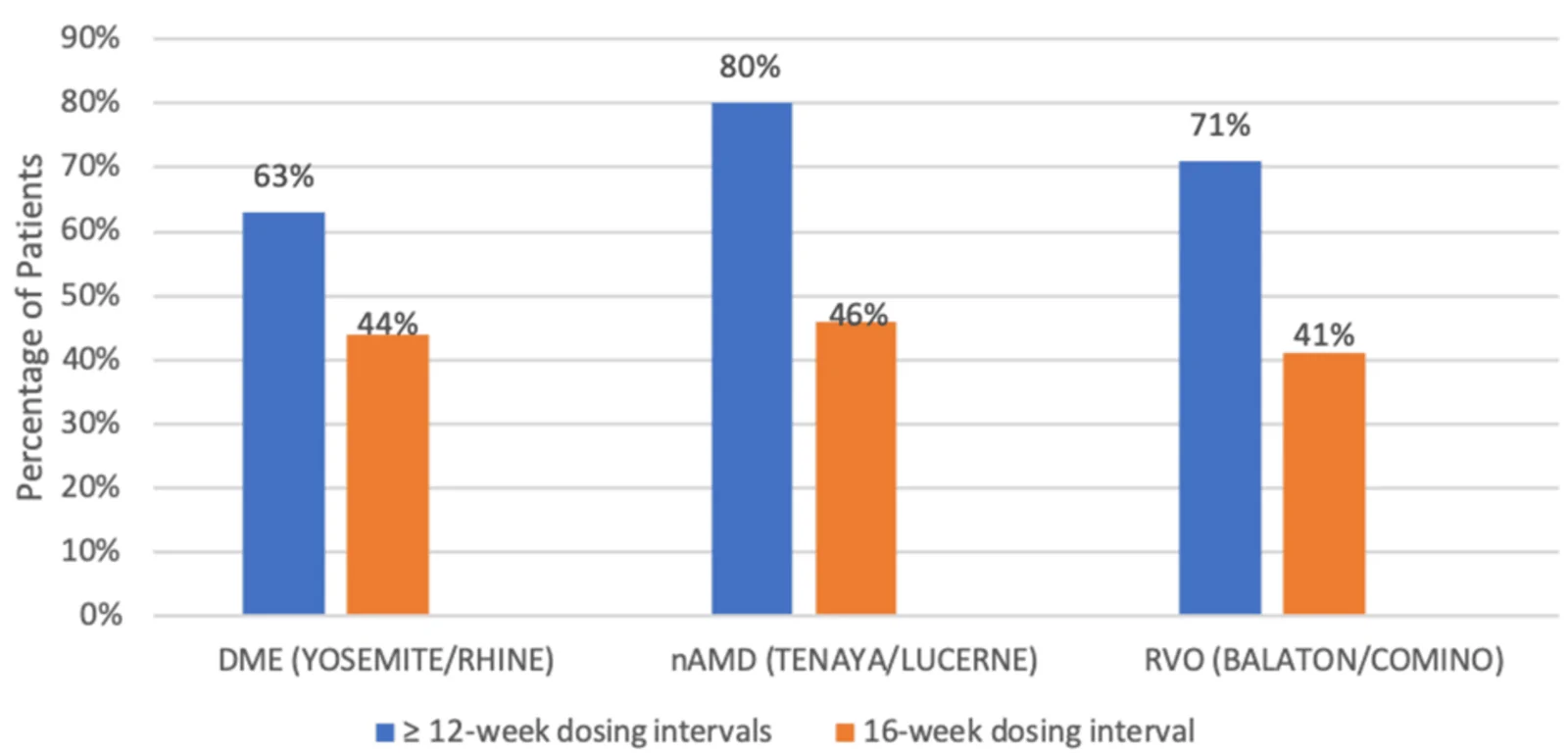
Extended dosing intervals achieved with faricimab across diabetic macular edema, neovascular age-related macular degeneration, and retinal vein occlusion Data were derived from the YOSEMITE/RHINE, TENAYA/LUCERNE, and BALATON/COMINO trials, respectively DME: diabetic macular edema; nAMD: neovascular age-related macular degeneration; RVO: retinal vein occlusion Source: [7,32,37]

In nAMD, Ang-2 and VEGF-A are both found within choroidal neovascular membranes, which contributes to vessel immaturity and leakage. The phase 3 TENAYA and LUCERNE trials compared faricimab, which could be dosed as infrequently as every 16 weeks, to aflibercept given every eight weeks. Over a two-year period, faricimab has achieved the primary endpoint of noninferior improvement in BCVA [32]. Treatment durability was a key secondary focus; in the combined analysis at week 48, more than 79% of patients on faricimab were on a 12- or 16-week dosing schedule, with over 45% reaching the full 16-week interval [33]. This extended interval outcome was predicted by the earlier STAIRWAY phase 2 trial, where 72% of patients maintained dosing every 16 weeks after initial monthly loading doses [27]. During anatomical evaluation, with the fixed dosing period, faricimab produced rapid reductions in CST similar to aflibercept [20]. The distribution of these achieved dosing intervals is summarized in Figure 1. Evidence from clinical practice in patients switched to faricimab from other anti-VEGF drugs (often due to persistent fluid or frequent need for injections) shows anatomical and functional improvements, including reductions in CST and stabilization of visual acuity [34-36].

Evidence from clinical practice in patients switched to faricimab from other anti-VEGF drugs (often due to persistent fluid or frequent need for injections) shows anatomical improvement and vision stabilization [34-36]. Therefore, faricimab achieves visual results similar to standard therapy while offering a clinically meaningful reduction in injection frequency for many nAMD patients.

Faricimab's greater anatomical responses were accompanied by favorable functional outcomes, including BCVA results, demonstrating its dual Ang-2 and VEGF-A suppression mechanism. In the YOSEMITE and RHINE phase 3 trials for DME, faricimab produced greater reductions in CST than aflibercept, with mean between-group differences of approximately 25-35 μm at several time points [29]. Faricimab also increased the rate of complete macular leakage resolution on fluorescein angiography [8].

In nAMD, the TENAYA and LUCERNE trials found comparable CST reductions, intraretinal fluid resolution, and subretinal fluid clearance between faricimab (up to every 16 weeks) and aflibercept (every eight weeks) during fixed-dosing phases [20]. The anatomical findings of the studies are summarized in Table 1.

**Table 1:** Comparative anatomic efficacy of faricimab and aflibercept in diabetic macular edema and neovascular age-related macular degeneration Findings were obtained from analyses of the YOSEMITE/RHINE and TENAYA/LUCERNE trials DME: diabetic macular edema; FA: fluorescein angiography; nAMD: neovascular age-related macular degeneration Source: [8,20,29]

Anatomic parameter	Favors faricimab	No difference	Favors aflibercept	Key quantitative findings
Central subfield thickness reduction (DME)	YOSEMITE/RHINE, 2 years [29]	-	Additional ~25-35 µm reduction vs. aflibercept	Approximately 25-35 μm greater reduction with faricimab than with aflibercept at several time points [29]
Complete leakage resolution on FA (DME)	YOSEMITE/RHINE, 1 year [8]	-	15%-20% more patients leak (compared to faricimab)	Complete absence of leakage was observed more frequently with faricimab than with aflibercept [8]
Intraretinal fluid resolution (nAMD)	-	TENAYA/LUCERNE, fixed-dosing period [20]	Comparable rates	Comparable rates
Subretinal fluid resolution (nAMD)	-	TENAYA/LUCERNE, fixed-dosing period [20]	Comparable rates	Comparable rates

These long-lasting effects supported greater vascular maturation from dual pathway inhibition [20]. More effectively than aflibercept in DME, faricimab reduces hyperreflective foci on optical coherence tomography, indicators of retinal inflammation, which is consistent with Ang-2's function in leukocyte adherence and cytokine production [38,39]. Clinical practice data confirms fluid resolution across indications and sustained CST improvements (e.g., -48.7 to -50.7 μm after seven injections) [30].

Retinal Vein Occlusion

Macular edema is caused by RVO, which is primarily VEGF-A-mediated. However, the Ang-2 pathway also contributes to the associated vascular leakage and inflammation following the occlusion. The efficacy of faricimab, which inhibits both pathways, was observed in phase 3 BALATON (for branch RVO) and COMINO (for central RVO) trials. At the 24-week primary period, monthly faricimab shows noninferior BCVA gains compared with monthly aflibercept [40]. Following this, a treat-and-extend period through week 72, over 70% of patients on faricimab achieved dosing intervals of 12 weeks (about three months) or longer, with more than 40% reaching the maximum 16-week interval [37]. This shows that after an initial period of monthly injections to control edema, faricimab can maintain therapeutic efficacy with a reduced treatment frequency. Previous studies from clinical practice support the long-lasting efficacy observed [41].

Supporting these trial outcomes, a recent real-world study investigated faricimab in patients with RVO who had shown a poor response to previous anti-VEGF therapies [42]. Following a structured four-month loading phase, these patients showed improvements in BCVA and reductions in central retinal thickness, with many achieving resolution of intraretinal fluid on optical coherence tomography. These results align with the idea that faricimab can be effective even in cases resistant to other anti-VEGF agents, supporting its potential as a durable and adaptable treatment option for RVO.

Hence, evidence from both controlled clinical trials and real-world practice proves that faricimab effectively maintains visual and anatomical benefits for patients with RVO-related macular edema while reducing the associated treatment burden.

Polypoidal Choroidal Vasculopathy

PCV is defined by polypoidal choroidal lesions and a branching vascular network, with a heterogeneous pathophysiology involving VEGF and inflammatory pathways. The dual-inhibition mechanism of faricimab provides a strong basis for use in PCV, which works on both angiogenesis and vascular instability.

Recent real-world and early clinical studies have begun to evaluate faricimab specifically in PCV patients. Ibrahim et al. reported that switching patients with nAMD and PCV to faricimab resulted in improvements in BCVA and reductions in CST over short-term follow-up [43]. Also, Arnold-Vangsted et al. showed that faricimab led to a reduction in polypoidal lesions in both treatment-naïve patients and those who had received prior therapy for PCV, while showing a more preferred safety profile [44].

Further studies have supported these findings, demonstrating favorable anatomical responses and fluid reduction following faricimab treatment, with some patients also achieving extension of treatment intervals, potentially reducing injection burden in PCV [43,44]. While these early data are promising, prospective, controlled trials comparing faricimab to established therapies that are required, including anti-VEGF monotherapy and combination therapy with photodynamic therapy, are needed to determine optimal dosing and long-term efficacy.

Faricimab shows potential as a therapeutic option for PCV and also provides dual pathway inhibition that may enhance polyp-reducing rate and improve macular anatomy while potentially reducing treatment frequency. More investigation into PCV-specific trials is needed to confirm the exact clinical role.

Safety and tolerability

**Figure 2:**
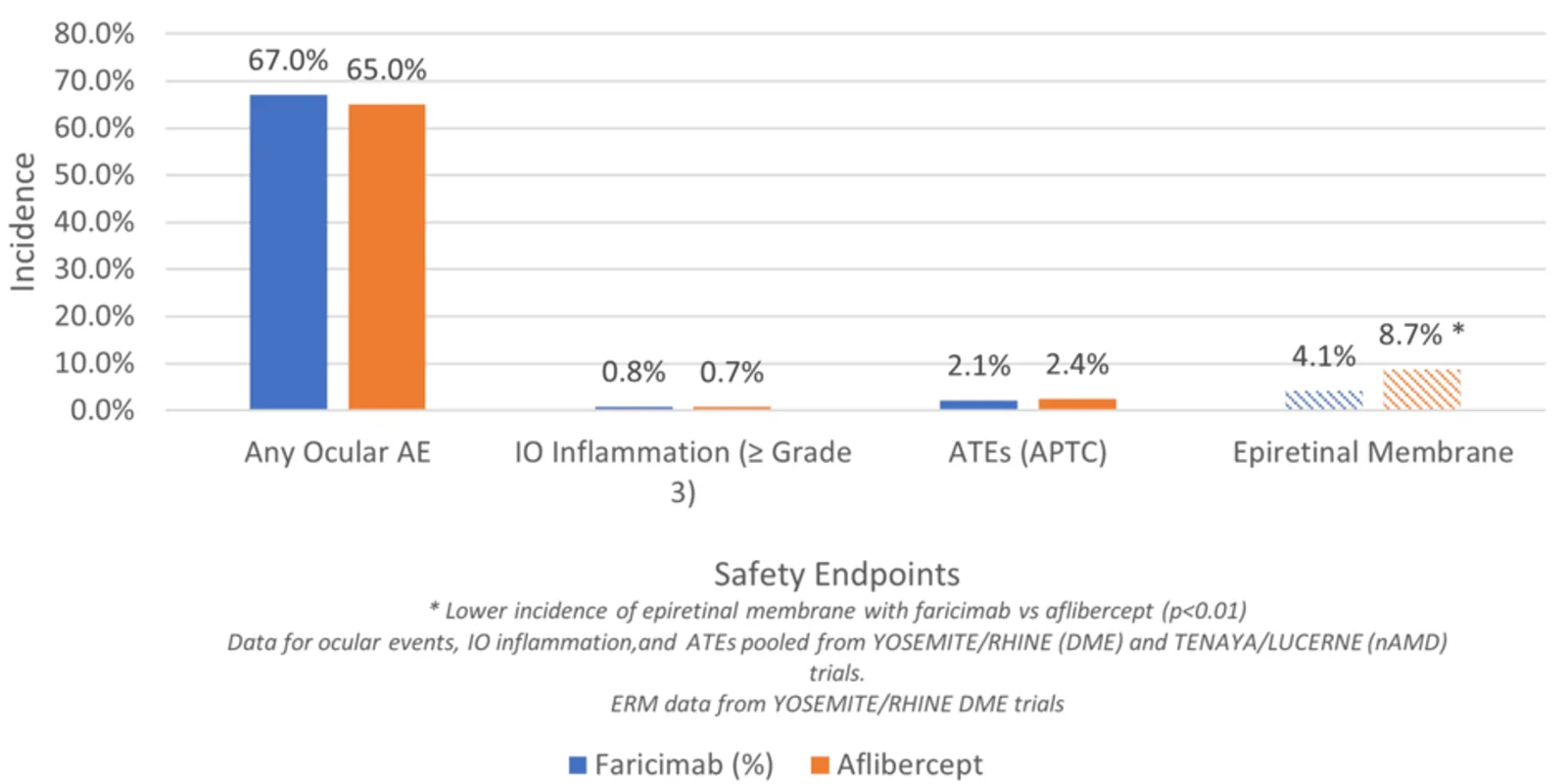
Comparative safety profile of faricimab and aflibercept in phase 3 clinical trials Ocular adverse event, intraocular inflammation, and arterial thromboembolic event data were derived from the YOSEMITE/RHINE and TENAYA/LUCERNE trials; epiretinal membrane data were derived from the YOSEMITE/RHINE trials AE: adverse event; IO: intraocular; ATE: arterial thromboembolic event; APTC: Antiplatelet Trialists’ Collaboration; ERM: epiretinal membrane; DME: diabetic macular edema; nAMD: neovascular age-related macular degeneration Source: [7,24,25,42]

Faricimab demonstrates a favorable and well-characterized safety profile across retinal disorders, distinguishing it from previous anti-VEGF monotherapies. As mentioned previously, phase 3 trials investigating faricimab for DME and nAMD had comparable incidence and severity of ocular and systemic adverse events to other VEGF inhibitors, with no new safety signals identified, as shown in Figure 2 [43,45].

In particular, common ocular adverse events included conjunctival hemorrhage, eye pain, and vitreous floaters occurring in 1%-5% of patients, while mild intraocular inflammation was observed in a small proportion [24,25,37]. Systemic adverse events, including arterial thromboembolic events, were rare and balanced across treatment arms [43,46]. Collectively, these data show that faricimab has a unique safety profile, as it lowers rates of epiretinal membrane formation and intraocular inflammation that have been reported compared with some other anti-VEGF agents [3,45,47]. The incidence of adverse effects with faricimab could theoretically be lower given its extended treatment durability, allowing for fewer injections while maintaining efficiency and potentially reducing the cumulative risk associated with frequent intravitreal procedures such as endophthalmitis or treatment burden [7,27,36]. Real-world evidence further supports its safety in patients with poor response or intolerance to prior therapies without an increased risk of intraocular inflammation, which is a particular concern observed with some other newer agents [44,47]. Systemic safety remains reassuring with no imbalance in arterial thromboembolic events reported. Pharmacodynamic studies suggest that faricimab’s dual inhibition does not induce a concerning systemic counterregulatory response [43,46]. In conclusion, faricimab offers an effective treatment with a safety profile consistent with established anti-VEGF therapies that enhances the potential for improved anatomical outcomes and reduced treatment frequency. The prolonged effects of faricimab may allow for fewer intravitreal injections, which can potentially reduce the overall treatment burden, including injection-related complications and clinic visits [7,27,36]. Real-world evidence further supports its safety in patients who shifted from other anti-VEGF therapies, including those with poor response or intolerance, without increased risk of intraocular inflammation [44,47]. Systemic safety is favorable, with arterial thromboembolic events occurring at rates comparable to other anti-VEGF agents [43,46]. Pharmacodynamic studies also support that dual inhibition of VEGF-A and Ang-2 does not induce systemic counterregulatory responses. Hence, faricimab shows a safety profile consistent with the current anti-VEGF therapies while providing the potential for reduced injection frequency and improved anatomical results.

## Conclusions

Faricimab represents a significant evolution in the treatment of retinal neovascular diseases, including nAMD and PCV, emerging from the growing understanding of the intertwined roles of Ang-2 and VEGF in the pathophysiology of retinal diseases and faricimab’s dual inhibition. This paper discusses the role of faricimab and its therapeutic impact in the management of nAMD, DME, and more retinal conditions. Collectively, the literature suggests that faricimab achieves visual outcomes comparable with standard anti-VEGF monotherapies, while offering significantly greater treatment durability and favorable anatomical outcomes. Across multiple trials, faricimab’s ability to extend therapeutic dosing to 12-16 weeks without compromising efficacy or outcomes is clinically substantial, as it reduces treatment burden, which remains a challenge in treating and managing retinal diseases. This significant discovery facilitates improved long-term adherence to medical regimens, particularly among patients who find frequent injections challenging.

Despite the advantages faricimab offers, it still presents certain limitations. Long-term real-world findings are still emerging, particularly in the context of chronic conditions like retinal vascular diseases, increasing the need to collect more data over longer periods to strongly confirm the associated advantages. Furthermore, the role of faricimab in treatment-resistant diseases and the possible integration of faricimab with other agents to achieve better outcomes warrant further investigation. Faricimab is poised to play an increasingly important role in the management of retinal and choroidal vascular diseases, as ongoing studies further clarify its long-term performance to optimize its clinical effectiveness.
